# When Words Shift: Age and Language of Elicitation Influence Syntagmatic-Paradigmatic Shifts in Bilingual Children

**DOI:** 10.3390/bs15121632

**Published:** 2025-11-27

**Authors:** Reinaldo Cabrera Pérez, Amy S. Pratt, Ashley M. Sanabria, Elizabeth D. Peña

**Affiliations:** 1 School of Education, University of California, Irvine, CA 92697, USA; 2 Department of Communication Sciences and Disorders, University of Cincinnati, Cincinnati, OH 45229, USA; 3 School of Speech, Language, and Hearing Sciences, San Diego State University, San Diego, CA 92182, USA; aadams2@sdsu.edu

**Keywords:** semantic development, multi/bilingualism, bilingual education, syntagmatic-paradigmatic shift, cognitive interviews

## Abstract

The shift from syntagmatic to paradigmatic associations is a developmental process occurring from approximately the ages of six to nine years and plays an important role in language development. Syntagmatic relationships refer to words that co-occur due to their mutual dependency connection (e.g., “The dog barks”). Paradigmatic relationships are words within the same category (e.g., cat, kitten). In Study 1, we tested 244 Spanish-English bilingual children in grades 1 to 3 (*M* age = 7.87 years, 54.5% female) enrolled in dual language programs in California, USA. Children completed a matching task in both English and Spanish featuring both syntagmatic and paradigmatic lexical associations. Results showed significantly higher accuracy for older students than for younger students, higher accuracy in English than in Spanish for both paradigmatic and syntagmatic associations, and higher accuracy in paradigmatic associations in English and syntagmatic associations in Spanish. In Study 2, we conducted cognitive interviews with a separate sample of 13 Spanish-English bilingual children (*M* age = 8.96 years, 46.15% female) to explore how they reasoned through their word pair choices when completing the task. Children primarily relied on paradigmatic associations, using strategies like synonymy, antonymy, and category overlap, while also employing syntagmatic associations and thematic relatedness as less frequent but important reasoning strategies. Implications for early language development are discussed.

## 1. Introduction

As children progress through school, they face increasing academic demands to define words and incorporate them into both spoken and written discourse. To meet these demands, children must develop an in-depth understanding of the lexicon, including categorical and functional attributes, grammatical classifications, and contextual sociolinguistic use. As their vocabulary grows, children also develop more efficient strategies for acquiring and organizing new words, moving from storing isolated items to building interconnected semantic networks that support retrieval, extension, and flexible use (Sheng et al., 2006, 2011). In early development, these networks are shaped primarily by experiential learning, resulting in syntagmatic associations. Syntagmatic associations link words based on their sequential syntactic role, typically forming connections between a subject and verb (e.g., associating “dog” with “barks”). As language knowledge consolidates, children begin to form paradigmatic associations, organizing words by deeper meaning and category, such as connecting “cat” with “kitten” as animals (V. S. Cronin, 2002; Francis, 1972; Nelson, 1977; Nissen & Henriksen, 2006; Wojcik & Kandhadai, 2020). This developmental phase, known as the syntagmatic-paradigmatic shift, typically occurs between the ages of six to nine among monolingual speakers (Brown & Berko, 1960; Entwisle, 1966; Ervin, 1961; Francis, 1972; McNeill, 1966) and reflects both cognitive and contextual influences.

For bilinguals, examining this shift is crucial for understanding how semantic organization develops in each of children’s languages, and whether patterns observed in monolinguals emerge similarly or differently when vocabulary growth and exposure vary across contexts. Such insights have broader implications for bilingual language development, as well-organized semantic networks in both languages support communication, academic language use, and cross-language transfer. Importantly, this period also coincides with increased exposure to school-based language practices and, for many bilingual children in US contexts, a shift in language dominance from Spanish to English (MacLeod et al., 2019; Welsh & Hoff, 2021). As such, the reorganization of the lexicon in bilinguals likely reflects the combined effects of maturation, formal schooling, and evolving language experience. In particular, the transition from a primarily oral, home-based language context to a school-based language context introduces children to literacy practices, academic vocabulary, and more decontextualized language use, which are expected to play a role in shaping the nature and timing of the syntagmatic-paradigmatic shift.

This shift in lexical organization becomes especially important as children encounter more complex academic language demands including reading and writing. Schooling presents a new context as children apply their lexical skills to the process of learning to read and write. Meta-analyses suggest that strong lexical skills are foundational for literacy development, as vocabulary knowledge is closely linked to reading comprehension outcomes in monolinguals (Elleman et al., 2009) and bilinguals (Zhang & Zhang, 2022).

### 1.1. The Syntagmatic-Paradigmatic Shift in Monolingual and Bilingual Speakers

While young children’s semantic networks include paradigmatic links early on, the strength and frequency of these links become more adult-like with age. In a free association study with 60 monolingual English-speaking children (ages 3–8), Wojcik and Kandhadai (2020) found that paradigmatic associations were present even in the youngest group and increased steadily: 31% at ages 3–5, 52% at ages 6–8, and 69% in adults. The prevalence of paradigmatic responses is also shaped by vocabulary factors. Low-frequency words tend to elicit syntagmatic or even phonological associations (Namei, 2004), and nouns tend to prompt paradigmatic responses earlier than other parts of speech (V. S. Cronin, 2002; Entwisle, 1966; Nissen & Henriksen, 2006; Rapp, 2002). Cross-language research indicates that language structure, cultural factors, and task modality further influence association patterns. For instance, Greek-English (Mattheoudakis, 2011) and Danish-English (Nissen & Henriksen, 2006) bilinguals produced more syntagmatic responses than English speakers, in part due to task demands such as written format or multiple-response requirements, which tend to favor chaining effects (Clark, 1993).

For bilinguals, the developmental shift from syntagmatic (event/script-based) to paradigmatic (taxonomic) relations parallels broader growth in grammar and phonology, as children move from context-bound, prosody-reliant utterances to more abstract, morphosyntactically complex language (Tomasello & Brooks, 2016; Hoff, 2015; Sheng et al., 2006; Simon-Cereijido & Méndez, 2018). The Revised Hierarchical Model (RHM; Kroll & Stewart, 1994; Kroll et al., 2010) provides a useful framework for interpreting these patterns, positing a shared conceptual system with language-specific lexicons whose links strengthen differently depending on age of exposure, proficiency, and dominance. In children, these links can develop simultaneously or sequentially across languages depending on input variation, quantity, and the contexts in which each language is used (Paradis, 2010; Bedore & Peña, 2008).

Empirical work demonstrates both shared and asymmetric developmental patterns. In Spanish–English bilinguals, Peña et al. (2002) found that younger children (mean age 5;1) generated similar numbers of responses to slot-filler (syntagmatic) and taxonomic (paradigmatic) prompts, whereas older children (mean age 6;5) produced more paradigmatic responses, suggesting a restructuring toward category-based organization. Sheng et al. (2006) reported that Mandarin–English bilinguals produced comparable numbers of paradigmatic responses in both languages, while Sheng et al. (2013) showed that vocabulary size and exposure shape the shift from syntagmatic (e.g., “eat” for “food”) to paradigmatic (e.g., “fruit” for “food”) associations in Spanish–English bilinguals. Other studies note that morphosyntactic and phonological development may progress more rapidly in the dominant or instructionally supported language (Fabiano-Smith & Goldstein, 2010), potentially leading to earlier semantic reorganization in that language. Parallel patterns have been observed in ASL–English bilinguals (Mann et al., 2016), underscoring that the shift is modality-independent.

How bilingual children comprehend, evaluate, and navigate cross-language lexical relations remain underexplored. As a result of a primary focus on lexical production, comprehension has been understudied, specifically how individuals recognize and process relationships between words. This gap limits our understanding of syntagmatic and paradigmatic lexical organization during language comprehension rather than production. One key aspect of comprehension that warrants further attention is lexical choice, the process by which children evaluate and select words based on meaning, context, and task demands. Unlike free word association, which reflects spontaneous access, lexical choice involves more reflective processing and inhibition of less relevant responses. This skill is especially important for bilingual children, who must navigate competing associations across two languages (Sheng et al., 2013; Simon-Cereijido et al., 2020). Studying how bilingual children make these lexical choices can shed light on the cognitive mechanisms that support semantic development and cross-language meaning-making.

### 1.2. Schooling, Literacy, and Lexical Organization

Reading proficiency, which encompasses decoding, fluency, and comprehension, plays an important role in vocabulary expansion and grammatical complexity (García & Cain, 2014; Liu & Saad, 2025; Tong et al., 2025) and even alters lexical activation within the speech recognition pathway (Schild et al., 2011). Thus, as children become literate, reading begins to support and enhance language growth. This developmental shift illustrates the reciprocity between language and reading, each skill reinforcing and shaping the other over time. Bilingual children face the unique challenge of developing verbal and written language skills across two linguistic systems, requiring them to navigate complex lexical associations within and across both languages, orally and in writing.

While most vocabulary studies involving bilingual children have focused on oral vocabulary breadth (i.e., the number of words a child knows; Bialystok, 2007; V. S. Cronin, 2002; Nissen & Henriksen, 2006), less attention has been given to vocabulary depth. Vocabulary depth refers to semantic richness, conceptual understanding, and the ability to form meaningful connections between words. Consequently, the ways in which bilingual children develop deeper lexical knowledge and semantic mapping remain understudied. The present study explores how Spanish-English bilingual children enrolled in dual-language programs navigate these syntagmatic-paradigmatic shifts across both of their languages. While most studies of the syntagmatic-paradigmatic shift focus on monolingual children, there are some emerging studies of bilinguals.

Schooling and literacy have been identified as key drivers of the syntagmatic–paradigmatic shift. Direct instruction, exposure to academic language, and reading acquisition foster children’s ability to organize words by category rather than sequential association (V. S. Cronin, 2002; V. Cronin et al., 1986; Leider et al., 2013; Mirman et al., 2017; Proctor et al., 2012). In particular, reading proficiency is closely linked to the prevalence of paradigmatic responses, suggesting that literacy promotes not only vocabulary growth but also deeper conceptual restructuring of the lexicon. Together, these findings indicate that the syntagmatic-paradigmatic shift reflects both cognitive maturation and the demands of formal schooling, providing an important context for examining bilingual children’s development. Beyond typical developmental trajectories, it is also important to consider learners with diverse developmental profiles. A recent systematic review of educational interventions by Andreou and Argatzopoulou (2025) reviewed how children with neurodevelopmental conditions (i.e., autism, attention-deficit/hyperactivity disorder, intellectual disability) acquire foreign languages, showing that adapted instructional methods, including gamified and visual supports, can enhance vocabulary learning and conceptual understanding. Similarly, research on vocabulary interventions for emergent bilinguals with, or at risk for, language impairment (Bedore et al., 2020; Lugo-Neris et al., 2015; Nair et al., 2023; Restrepo et al., 2013) has demonstrated measurable gains in vocabulary and grammar over relatively short intervention periods.

Building on this foundation, bilingual children follow a similar overall trajectory as monolinguals in shifting from syntagmatic to paradigmatic lexical associations, but the timing and pattern of this shift may vary across languages and individuals. For instance, children with consistent exposure to print and academic vocabulary in English may develop paradigmatic associations more robustly in that language, while continuing to rely on syntagmatic associations in Spanish if exposure remains largely oral and context-bound. Children in dual language programs or those who broker meaning across languages may develop more flexible and interconnected lexical networks, potentially accelerating the shift in one or both languages.

In order to address these questions two studies were undertaken, Study 1 aimed to examine lexical-semantic development in Spanish-English bilingual children by assessing the accuracy of syntagmatic and paradigmatic associations across languages and analyzing how performance varies by grade level. Study 2 focused on understanding the reasoning behind children’s lexical choices to better understand how they interpret meaning in a bilingual context, and the cognitive mechanism guiding their semantic decisions.

## 2. Study 1: Lexical Association Accuracy Across Languages and Grades

Study 1 examined syntagmatic and paradigmatic association accuracy in Spanish-English bilingual children using a novel bilingual word-matching task. A word-matching task was selected for its ability to capture children’s semantic organization through observable accuracy in selecting related lexical items. The study explores how accuracy varies across languages (Spanish and English), and grade levels (first through third). We hypothesized that children would show higher accuracy in their strongest language and perform better on paradigmatic associations across both languages (Nelson, 1977; Sheng et al., 2013). We also expect that accuracy, particularly for paradigmatic associations, will increase with grade level, consistent with developmental trends in semantic organization (V. S. Cronin, 2002; Sheng et al., 2013; Wojcik & Kandhadai, 2020; Costa et al., 2023; Lemke et al., 2021).

We further anticipated that these differences would reflect not only quantitative variation in vocabulary knowledge but also qualitative differences in how lexical relationships are structured and accessed. Specifically, we expected paradigmatic associations to be more developed in English due to its use in academic and literate contexts, whereas Spanish, which is used more frequently at home; might support stronger syntagmatic associations tied to everyday routines. Over time, the less dominant language may catch up, particularly for children with balanced exposure or dual-language instruction. Thus, we predicted that each language’s context of use, not just dominance, would shape both the pace and nature of the syntagmatic-paradigmatic shift, revealing how children use words, not just whether they know them.

### 2.1. Method

#### 2.1.1. Participants

A total of 244 bilingual Spanish-English children, ages (*M* = 7.87, range = 6;10–10;0), were selected from a pool of 474 participants from the pilot phase of a multi-year project aimed at developing Spanish and English across different instructional settings (i.e., dual-language). In these programs, instruction began with approximately 90% Spanish and 10% English. As students progressed through the grades, the amount of English gradually increased; however, from first through third grade, most instruction remained in Spanish. The majority of the sample spoke Mexican Spanish, the predominant dialect represented in the study. Participant descriptive statistics are presented in Table 1. Students were eligible to participate if they met two criteria: **(a)** they were enrolled in first through third grade, and **(b)** they were regularly exposed to both Spanish and English.

Recruitment took place during the 2021–2022 and 2022–2023 school years. A total of 14 classrooms were recruited from 6 schools in Southern California. Participants’ Spanish-English proficiency was determined based on their program enrollment in their bilingual program. The sample included a heterogeneous group of students, with some coming from Spanish-speaking homes and others learning Spanish primarily through school instruction.

#### 2.1.2. Measures

##### Palabras en Parejas (PEP)

**Figure 1:**
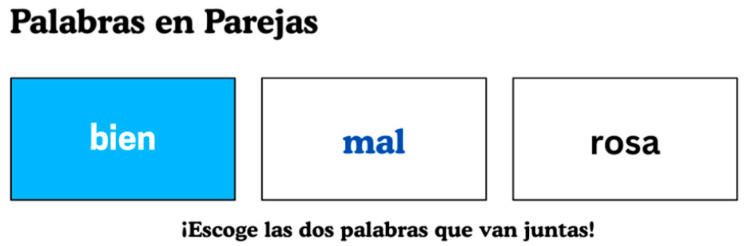
Palabras en parejas (PEP) task depiction.

Palabras en Parejas (words in pairs; PEP: Sanabria & Peña, 2021) is a self-paced experimental Spanish-language task designed to assess semantic skills in students across different grade levels. Instructions are given in Spanish via computer audio, and children respond to items displayed on a screen. Figure 1 presents a visual representation of PEP and how it is displayed. Each item consists of an anchor word, a target word, and a distractor. Approximately 150 items were developed to capture a broad spectrum of semantic knowledge. To ensure validity, PEP items were created independently rather than translated from an English version (Peña, 2007). Specifically, items were derived from Common Core State Standards (California Department of Education, 2012) and grade-level texts. In addition, the frequency of occurrence for each item was measured to ensure that words were appropriately representative of students’ linguistic exposure. This systematic approach resulted in a final word list tailored to each grade level’s linguistic proficiency and cognitive development.

Word association items were organized into grade-level testlets. There were two testlets for each grade level, each containing 35 items (including six anchor items). Testlets were selected to evaluate students’ grade-level proficiency as well as performance on items from adjacent grade levels. For example, first graders were presented with kindergarten, first-, and second-grade testlets, while third graders completed first-, second-, and third-grade testlets. Anchor words were categorized into three tiers based on their frequency and context of use. Target words were selected based on their semantic relationship to the anchor word. Appendix A presents the word selection design, including examples for each category.

##### Word Match Game (WMG)

The Word Match Game (WMG: Connor et al., 2022) is an adaptive English-language task designed to evaluate students’ semantic knowledge through a word-matching task. Similarly to Palabras en Parejas, this task measures students’ ability to recognize meaningful relationships between words. The WMG has well-established psychometric properties and has been validated with subtests from the Woodcock-Johnson III Test of Achievement (Woodcock et al., 2001) and the Gates-MacGinitie Reading Test (MacGinitie et al., 2000). This validation allows for the use of age- and grade-equivalent scores as dependent variables. For a comprehensive overview of the development of WMG, see Connor et al. (2022).

To ensure cross-language equivalence, we examined lexical frequency and item difficulty in Spanish and English. For PEP, we determined the level of difficulty (easy, medium, hard) and vocabulary appropriateness in consultation with biliteracy development specialists. Lexical frequency was calculated for each word, and items were scored for phonological and orthographic length as well as cognate overlap (including initial letter, syllable count, consonant and vowel overlap). For WMG, which was normed and validated in previous studies (Connor et al., 2022), we accounted for level of difficulty and lexical frequency in the analysis.

#### 2.1.3. Procedures

Children completed the assessments individually in a whole-class setting, with research assistants providing support as needed. All tasks were self-paced, with instructions delivered via computer audio on Chromebooks or tablets. Children wore headphones to ensure a clear audio presentation as they listened to three words, each indicated by a blue box during playback, before selecting the two words that best go together. English and Spanish tasks were administered on separate days to minimize language co-activation and reduce fatigue, with responses recorded automatically. Children were shown different items, which were classified to control for variations in association item type and difficulty across grade levels and vocabulary knowledge. Spanish and English sessions were counterbalanced at the classroom level so that each classroom completed one language version first (e.g., all students began with English or Spanish), minimizing order effects. Children did not receive feedback during task administration to avoid learning effects between sessions. Using a predetermined key, participants’ responses were automatically scored by the testing platform as “0” for incorrect answers and “1” for correct answers.

#### 2.1.4. Coding

We categorized the associations between anchor words and target words for each item in the PEP and WMG tasks as either syntagmatic or paradigmatic based on a predefined set of definitions and classification criteria informed by Söderman (1993) and Wolter (2001). Syntagmatic relations were identified based on three primary characteristics. (1) Syntactic adjacency are words that appear next to each other in a sentence or phrase. For example, in the sentence *“He drank a glass of milk,”* the words *glass* and *milk* are syntactically adjacent and commonly used together. (2) Thematic connection describes words that share a contextual or thematic link that enhances meaning. In the phrase *“She bought some bread and butter,”* the words *bread* and *butter* are thematically related, as they are commonly associated with each other in the context of food. (3) Mutual dependency occurs when the meaning of one word relies on the presence of another. For example, in “*I want to drink water*,” the words *drink* and *water* exhibit mutual dependency, because the verb *drink* implies something is being consumed, while *water* is defined by being something that can be drunk.

Paradigmatic relations were classified according to three key properties. (1) Same grammatical class refers to words belonging to the same part of speech, such as nouns, verbs, or adjectives. For example, the verbs *start* and *finish* can substitute for each other while maintaining grammatical correctness in a sentence, as in *“He can start early”* and *“He can finish early.”* (2) Taxonomic category refers to words that belong to the same conceptual group at the same hierarchical level. For instance, *apple* and *banana* are both nouns that fall under the broader category of fruits. (3) Interchangeability describes words that can be substituted in a sentence without affecting grammatical structure, though their meanings may differ. For example, the adjectives *happy* and *joyful* are interchangeable in *“She feels happy today”* and *“She feels joyful today.”* To ensure a balanced representation of items, we also considered the distribution of syntagmatic and paradigmatic relationships. Items were assigned across categories to maintain variety in linguistic structures and word relationships. Coders were blind to item level of difficulty during the syntagmatic and paradigmatic classification.

#### 2.1.5. Inter-Rater Reliability

To assess inter-rater reliability, the first author and two research assistants independently categorized each item as syntagmatic or paradigmatic. After two rounds of coding, we achieved 91.25% agreement for Spanish and 90.43% for English. Any remaining discrepancies were resolved through discussion until full consensus was reached. In addition to categorization, we examined the overall distribution of items to ensure a consistent representation of both syntagmatic and paradigmatic relationships across tasks. PEP included 78 syntagmatic items and 78 paradigmatic items (e.g., a third grader saw 18 items for each), whereas the adaptive WMG included 97 syntagmatic items and 187 paradigmatic items (e.g., a third grader saw 4–9 items for each). The absolute lexical frequency for PEP ranged from 1 to 51 responses per value, whereas for WMG, each lexical item appeared uniquely (i.e., with a frequency of one).

### 2.2. Results

#### 2.2.1. Association Accuracy by Language and Grade Level

Descriptive analyses revealed that children were more accurate in English than in Spanish across both association types. In Spanish, accuracy was slightly higher for syntagmatic (*M* = 0.61, *SD* = 0.21) than paradigmatic associations (*M* = 0.58, *SD* = 0.19), and this difference was statistically significant, *t*(242) = −2.59, *p* = 0.010, *d* = 0.17. In contrast, children were more accurate on English paradigmatic associations (*M* = 0.82, *SD* = 0.13) than syntagmatic ones (*M* = 0.77, *SD* = 0.22), also yielding a statistically significant difference, *t*(243) = 3.30, *p* = 0.001, *d* = 0.21.

Accuracy increased with grade level in both languages. In Spanish, first-grade students demonstrated the lowest accuracy for both paradigmatic (*M* = 0.55, *SD* = 0.20) and syntagmatic associations (*M* = 0.55, *SD* = 0.20), followed by second graders (paradigmatic: *M* = 0.57, *SD* = 0.18; syntagmatic: *M* = 0.61, *SD* = 0.21). The highest Spanish scores were observed in third grade (paradigmatic: *M* = 0.63, *SD* = 0.18; syntagmatic: *M* = 0.70, *SD* = 0.20). A similar trend was observed in English. First graders scored *M* = 0.79 (*SD* = 0.14) for paradigmatic and *M* = 0.77 (*SD* = 0.22) for syntagmatic associations. Second-grade students showed a slight increase in paradigmatic accuracy (*M* = 0.82, *SD* = 0.12), though syntagmatic scores declined slightly (*M* = 0.73, *SD* = 0.24), a pattern likely reflecting age-related differences and the fact that they encountered more difficult items than first-grade students. By third grade, accuracy was highest for both association types (*M* = 0.87, *SD* = 0.12 for paradigmatic; *M* = 0.87, *SD* = 0.18 for syntagmatic). See Table 2 for a summary of the proportion of items by grade and language.

#### 2.2.2. Influence of Grade, Language, and Association Type on Accuracy

##### Spanish Accuracy (PEP)

We used a linear mixed-effects model to examine the effects of grade level (first, second, third), association type (syntagmatic vs. paradigmatic), and lexical frequency on Spanish accuracy scores (PEP). Fixed effects included all three predictors and their interactions, with a random intercept to account for individual variability.

The analysis revealed a significant main effect of grade level, *F*(2, 341.7) = 3.58, *p* = 0.032. Third graders (*M* = 0.67, *SE* = 0.04) scored significantly higher than first graders (*M* = 0.59, *SE* = 0.03), *b* = 0.08, 95% CI [0.01, 0.14], *p* = 0.03, while second graders did not differ from first graders, *b* = 0.01, 95% CI [−0.05, 0.06], *p* = 0.76. Association type was not a significant predictor of accuracy, *b* = 0.02, 95% CI [−0.03, 0.07], *p* = 0.48, nor did it interact with grade (all *ps* > 0.05), indicating similar performance across syntagmatic and paradigmatic associations. Lexical frequency also had no significant effect, *b* = −0.00, 95% CI [−0.00, 0.00], *p* = 0.13. Random effects indicated substantial individual variability (τ = 0.02), with an intraclass correlation coefficient (ICC) of 0.64. The model explained 5.7% of the variance with fixed effects alone (marginal coefficient of determination R^2^ = 0.06), and 65.8% when including both fixed and random effects (conditional R^2^ = 0.66).

##### English Accuracy (WMG)

A linear mixed-effects model revealed a significant main effect of grade level, *F*(2, 461.4) = 3.30, *p* = 0.04. Third graders (*M* = 0.79, *SE* = 0.02) outperformed first graders (*M* = 0.71, *SE* = 0.02), *b* = 0.07, 95% CI [0.01, 0.14], *p* = 0.02, while second graders did not differ significantly from first graders, *b* = 0.03, 95% CI [−0.02, 0.08], *p* = 0.22.

Association type had no overall effect on accuracy, *b* = −0.02, 95% CI [−0.07, 0.04], *p* = 0.55. However, a significant interaction emerged for second graders, who performed worse on syntagmatic than paradigmatic associations, *b* = −0.08, 95% CI [−0.14, −0.01], *p* = 0.020. This interaction was not significant for third graders, *b* = 0.02, 95% CI [−0.06, 0.10], *p* = 0.63.

Frequency was not a significant predictor, *b* = 0.00, 95% CI [−0.00, 0.00], *p* = 0.65. The model indicated moderate individual variability (τ = 0.01; ICC = 0.21), with 6.6% of variance explained by fixed effects (marginal R^2^ = 0.07) and 26.0% by the full model (conditional R^2^ = 0.26).

##### Combined Language Accuracy

We used a linear mixed-effects model to examine accuracy across grade level (first through third), language (English, Spanish), association item type (syntagmatic vs. paradigmatic), and word frequency, with random intercepts for students.

First-grade students scored an average of 0.79 on English paradigmatic items (intercept). Accuracy was significantly lower in Spanish than in English, *b* = −0.24, SE = 0.03, *p* < 0.001. Third graders outperformed first graders across conditions, *b* = 0.08, SE = 0.03, *p* = 0.03, while second graders did not differ significantly, *b* = 0.03, *p* = 0.25. There were no main effects of association item type (*b* = −0.02, *p* = 0.44) or frequency (*b* ≈ 0, *p* = 0.92).

**Figure 2:**
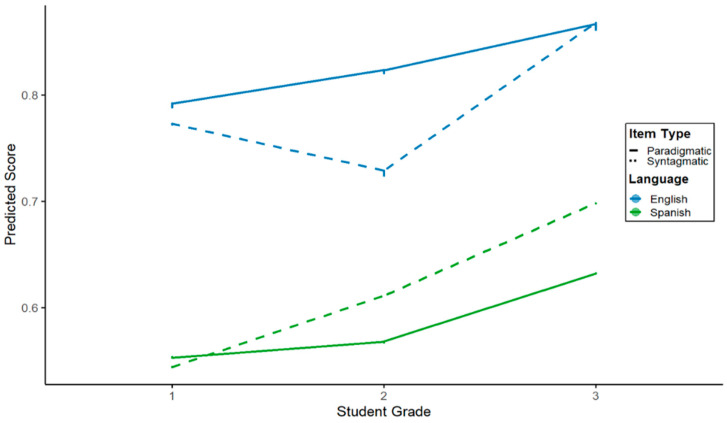
Interaction between student grade, language, and association item type.

A significant interaction between grade (second vs. first) and association item type, *b* = −0.08, *p* = 0.02, indicated that the syntagmatic–paradigmatic relationship in second grade differed from that of the first graders. This was further qualified by a three-way interaction among grade, language, and association item type, *b* = 0.13, *p* = 0.005, showing language-specific differences in association accuracy for second graders. No significant interactions were found for third grade or for language by association item type. Random effects showed substantial individual variability (ICC = 0.31). Fixed effects explained 26.6% of the variance (marginal R^2^ = 0.27), and the full model explained 49.2% (conditional R^2^ = 0.492). Figure 2 illustrates changes in accuracy across grades.

### 2.3. Interim Discussion

Findings from Study 1 supported expected developmental patterns, higher accuracy in English and increasing paradigmatic accuracy with age and schooling. Findings also revealed a key cross-language difference effect: while English association accuracy showed a stronger shift toward paradigmatic associations, Spanish association accuracy remained higher on syntagmatic items across grades. This differential pattern may reflect the qualitatively different contexts in which each language is used. One possible explanation is that English may be more present in the home environment for students who are simultaneously learning Spanish in school, leading to greater exposure to English paradigmatic structures. In addition, the observed difference could reflect the nature of the items themselves, as some may be more context-specific across languages, thereby eliciting higher accuracy on syntagmatic associations in Spanish (McMillen et al., 2022). The results align with the view that bilingual development is experience-dependent, with each language reflecting the functions and demands of its typical context (Paradis, 2010; Sheng et al., 2006, 2013). Rather than assuming a unified developmental trajectory across both languages, these findings demonstrate the need to consider language-specific pathways in semantic development.

## 3. Study 2: Lexical Selection Through Cognitive Interviewing

Findings from Study 1 provided patterns in bilingual children’s accuracy on lexical association tasks across Spanish and English. However, quantitative accuracy metrics alone offer limited insight into the cognitive and semantic strategies underlying children’s responses. To better understand how children make these lexical associations, Study 2 employed a cognitive interview methodology. Cognitive interviewing provides an opportunity to explore children’s thought processes during task performance, shedding light on their interpretation of lexical relationships and the reasoning behind their choices (Beatty & Willis, 2007; Brédart et al., 2014; Simon-Cereijido et al., 2020).

By eliciting children’s explanations, cognitive interviews allow researchers to investigate strategies such as mutual dependency, thematic or taxonomic relationships, and category flexibility. Cognitive interviewing, as described by Beatty and Willis (2007), is a flexible method that emphasizes children’s thought processes rather than merely their final responses, making it especially useful for uncovering how children interpret and reason through language tasks. This approach is particularly valuable when working with bilingual or culturally diverse populations, as it shifts focus from correctness to understanding—much like Ginsburg’s (1997) findings that children from diverse backgrounds performed better in clinical interviews than on traditional assessments. Rather than relying solely on standardized testing formats, cognitive interviews invite children to reflect on their associations and clarify their reasoning, revealing nuances in paradigmatic and syntagmatic thinking that may otherwise go unnoticed. The use of think-aloud protocols (Ericsson & Simon, 1980, 1993) and well-targeted probes (Foddy, 1998; Willis, 2005) further supports this process by facilitating insight into children’s internal categorization strategies, ultimately enhancing our understanding of their conceptual and lexical development.

Study 2 focused on Spanish-English bilingual children between the ages of seven and ten. Following completion of a lexical association task in both Spanish and English, children were asked to reflect on and explain their reasoning behind selected responses, irrespective of response accuracy. By integrating children’s qualitative explanations with their performance data, this study aims to provide a more nuanced understanding of how bilingual children form and organize lexical-semantic relationships across their two languages.

### 3.1. Method

#### 3.1.1. Participants

Inclusion criteria were consistent with Study 1. An independent sample of fourteen Spanish-English bilingual children were recruited from Southern California and Florida, with a final sample of 13 participants (*M* = 8.96 years, range = 7;1–10;3).^1^ Due to limited access to the original schools and the time elapsed since the initial data collection, it was not feasible to recruit participants from the Study 1 sample. The sample was 46.15% female and 53.85% male. Eleven children spoke Cuban-Spanish and two spoke Mexican-Spanish. Children were assessed in various settings, including community centers (e.g., libraries, after-school programs) and during home visits.

#### 3.1.2. Measures

Measures were consistent with Study 1, with the exception that the word-matching tasks were administered in a paper-based format in both Spanish and English, rather than computerized. This format was selected to align with the reflective nature of the cognitive interview and to minimize the likelihood of rapid or impulsive responses. To assess vocabulary knowledge, children completed a picture-based vocabulary selection task to establish baseline familiarity with the target words. A total of ten items (five Spanish, five English) for the cognitive interview were selected for inclusion on Study 2 based on item representativeness (i.e., syntagmatic and paradigmatic items) and item difficulty based on the responses of participants on sample Study 1.

#### 3.1.3. Procedures

**Figure 3:**
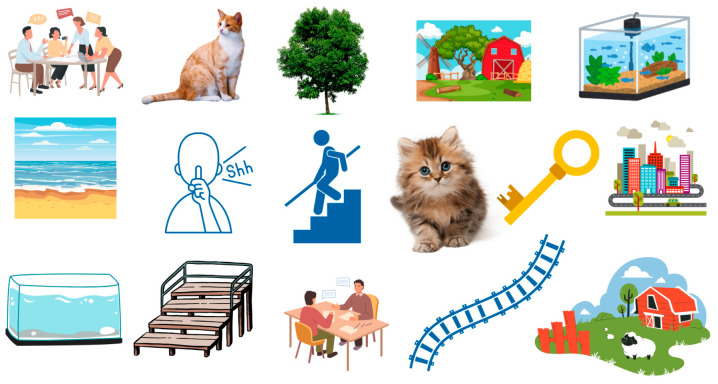
Sample English image items used to elicit target words.

Stimuli and procedures closely followed those of Study 1, with the primary difference being the use of paper-based word-matching tasks. To ensure child familiarity with the vocabulary concepts, children were presented with a sheet containing images and asked to identify the target word in the corresponding language (i.e., Spanish or English) prior to the cognitive interview (see Figure 3). Children then completed the word-matching task, with language order counterbalanced across participants. Following each item response, children were asked to explain their choice. For incorrect responses, children were prompted to elaborate on their reasoning to better understand potential syntagmatic-to-paradigmatic shifts in lexical associations. After completion of the cognitive interview, the first author recorded memos for each child about their testing session, such as non-verbal communication, additional information provided after interviews, and parental input.

#### 3.1.4. Coding and Analysis

We analyzed children’s verbal explanations to better understand the conceptual and semantic strategies underlying their lexical associations. Employing an inductive qualitative approach (Yin, 2014), we developed a codebook informed by both established definitions of syntagmatic and paradigmatic relationships (e.g., Söderman, 1993; Wolter, 2001) and patterns emerging directly from the data. The analytic process unfolded in three phases. First, we identified accurate responses to the word-matching tasks within the transcribed interview documents. In the second phase, we coded each response as correct or incorrect, generating inductive categories based on the nature of lexical relationships. These codes were defined and organized in a master Excel file according to thematic rationales (e.g., synonyms, antonyms, measurement). In the final phase, we conducted a more fine-grained analysis of lexical associations, classifying responses according to phonological, orthographic, and semantic features.

We used Sonix (2025) and Dedoose (2025) to manage interview transcription and analyze the data. Each explanation was coded for both response accuracy and the underlying reasoning, including thematic connections, mutual dependency, categorical relationships (e.g., taxonomic links), and instances of phonological or orthographic overlap. The first author and a trained research assistant independently coded all responses, achieving an initial interrater agreement of 91.47% and resolving discrepancies through discussion to reach full consensus. Finally, we calculated the proportion of each code type across languages and children to explore how bilingual children interpret word relationships.

### 3.2. Results

#### 3.2.1. Lexical Associations

Children demonstrated higher overall accuracy in Spanish (*M* = 0.85, *SD* = 0.25) than in English (*M* = 0.72, *SD* = 0.25), *t*(12) = −1.98, *p* = 0.07. When examining syntagmatic and paradigmatic performance, accuracy patterns varied by language and association type. Specifically, children showed greater accuracy on paradigmatic items in English (*M* = 0.85, *SD* = 0.31) than syntagmatic (*M* = 0.67, *SD* = 0.30), *t*(12) = 2.11, *p* = 0.05, while in Spanish, syntagmatic scores (*M* = 0.92, *SD* = 0.19) were higher than paradigmatic scores (*M* = 0.80, *SD* = 0.32), though the difference was not statistically significant, *t*(12) = −1.86, *p* = 0.09. These results partially align with Study 1. While children in Study 2 demonstrated overall higher accuracy in Spanish, contrasting with the English-dominant performance observed in Study 1, the cross-language pattern remained consistent, with greater paradigmatic accuracy in English and greater syntagmatic accuracy in Spanish. This suggests a stable association between language and association type across studies, despite differences in overall language dominance. These differences may also reflect variation in children’s backgrounds: whereas Study 1 included more English-dominant children from California, most children in Study 2 were from Florida and were more Spanish-dominant. Thus, while overall accuracy shifted with language dominance and location, the underlying pattern of semantic organization by language remained persisted. This reinforces the idea that semantic development in bilinguals is shaped not only by proficiency, but by the contextual roles and functional use of each language, supporting the broader claim that bilingual lexical organization follows language-specific, experience-dependent pathways.

In exploring lexical association strategies, analysis of code co-occurrence revealed that correct responses across children (*n* = 13) were more frequently supported by paradigmatic associations (*n* = 84) than by syntagmatic associations (*n* = 21). Thematic relatedness emerged as the most prevalent code across both correct and incorrect responses. In several cases, children explained their reasoning by referencing shared contexts or functions, often using richly detailed justifications.

For instance, one child provided a correct paradigmatic association when presented with the words *ranch*, *farm*, and *city*, selecting *ranch* and *farm* as the correct pair and explaining: *“Because a ranch has many different animals ranging from either a herding dog to the enormous cow we have today and a farm has many things like that and the only difference between them is that some ranches don’t have horses and some farms have horses.”* This explanation exemplifies a conceptual connection based on semantic similarity and category overlap (see Appendix B for additional examples of participant responses).

Conversely, some incorrect responses reflected a shift from paradigmatic to syntagmatic associations. One notable example involved the words *planta* (“plant”), *pollo* (“chicken”), and *arbusto* (“bush”), where a child incorrectly selected *planta* and *pollo*. Their explanation: *“Porque es como una comida [pollo] que tú puedes poner en una sartén y las plantas como una las hojas que le le puedes echar a la comida”* (“Because it’s like a food [chicken] you can put in a pan and the plants like the leaves you can add to the food”), demonstrates the use of thematic and functional associations rather than categorical similarity, contributing to the observed error.

Children also drew on a range of other associative mechanisms, including orthographic, phonological, semantic, and thematic relations. Paradigmatic responses frequently included associations based on synonymy, antonymy, and size comparisons, where “size” referred to physical dimensions such as body, area, or structure. In addition, some children supplemented their verbal responses with non-verbal gestures or expressions, further illustrating the multimodal nature of their cognitive and communicative strategies during the tasks.

In analyzing children’s responses, it is important to note the balance between paradigmatic and syntagmatic reasoning observed across the responses. Many children focused on paradigmatic relationships, identifying shared characteristics like size, color, or category (e.g., “gato” (cat) and “león” (lion) both being animals, “planta” (planta) and “arbusto” (bush) both being plants or foliage), while others emphasized syntagmatic relationships, explaining functional connections between words, such as “aquarium” and “tank” or “bleacher” and “rail,” based on how they function together in context. Interestingly, responses in English were often more detailed and nuanced than in Spanish, with children providing more extensive explanations for functional relationships. Some children shifted their reasoning depending on the task at hand, such as moving from focusing on rhyming components (e.g., “mal” and “rosa”) to functional relationships (e.g., “mal” and “rosa” based on the concept of decay). In reflecting on this, children tend to group words either by similarity or function, with language differences influencing the depth and complexity of their justifications. It is also possible that paradigmatic relationships were easier for children to explain, as they draw on more concrete, categorical knowledge, whereas syntagmatic relationships require contextual reasoning and are often more abstract. This may partly explain the greater elaboration and clarity in paradigmatic responses, especially in the language of instruction.

#### 3.2.2. Bilingual Language Profiles

Across the 13 cognitive interview memos, children exhibited diverse bilingual profiles shaped by factors such as language dominance, exposure, and individual reasoning styles. Spanish was often the dominant or preferred language for expression, even when English was used more frequently at school. Participants with stronger bilingual abilities (*n* = 5) provided elaborate justifications and showed flexibility in both paradigmatic and syntagmatic associations across both Spanish and English, whereas others (*n* = 3) gave shorter responses or relied more heavily on word appearance and concrete features. Ambiguity in certain items (e.g., “ranch,” “tank,” “rail”) elicited varied interpretations, demonstrating the influence of linguistic and cultural context. Many participants self-monitored, revised their responses, or verbalized their thought processes, revealing metalinguistic awareness. Differences in school versus home language use, as well as developmental and expressive language abilities, played key roles in how children understood and completed the tasks.

## 4. General Discussion

Spanish-English bilingual participants between age 6;1 and 10;0 completed word-matching tasks in both Spanish and English. We analyzed their responses to assess their syntagmatic and paradigmatic lexical associations. In Study 1, we examined children’s response accuracy across task language (i.e., Spanish, English), grade level (i.e., first through third), and word association type (i.e., syntagmatic, paradigmatic). Consistent with the syntagmatic-paradigmatic shift observed in previous research (e.g., V. S. Cronin, 2002; Mattheoudakis, 2011; Nelson, 1977; Nissen & Henriksen, 2006), results revealed developmental changes in lexical-semantic accuracy, with patterns differing by language and grade. In Study 2, a separate group of bilingual children (ages 7;1–10;3) participated in cognitive interviews designed to elicit the reasoning strategies underlying their lexical choices. These interviews provided rich, qualitative insight into the semantic processes children used, such as thematic links, mutual dependency, and categorical reasoning, offering a deeper understanding of the mechanisms driving observed performance patterns. Together, both studies demonstrated how bilingual children’s lexical organization is shaped by age, language dominance, and instructional context.

### 4.1. Developmental Patterns in Lexical Organization Across Grade Levels

Consistent with prior research (V. S. Cronin, 2002; Nelson, 1977; Peña et al., 2002, 2003; Sheng et al., 2006, 2013), the present studies confirmed that lexical organization becomes increasingly paradigmatic with age and academic experience. Overall, Study 1 results align with our expectations, showing developmental increases in accuracy that vary by language and association type, reflecting both proficiency and contextual use. Accuracy increased across grade levels, particularly for paradigmatic associations in English, the dominant language for most participants. This finding is aligned with developmental expectations and supports the idea that school-based literacy experiences, such as reading instruction and vocabulary exposure, facilitate the shift from syntagmatic to paradigmatic associations (V. S. Cronin, 2002; Wojcik & Kandhadai, 2020). However, the results also demonstrate that this shift is not uniform across languages. In Spanish, syntagmatic associations remained more accurate than paradigmatic ones, especially among younger students, suggesting that language dominance and exposure influence the rate and nature of this developmental shift. This raises an important question for bilingual development: do children need to progress through the syntagmatic stage in each language separately, or can paradigmatic reasoning established in one language bootstrap development in the other? Our findings may suggest the latter could be possible, as conceptual knowledge appears to transfer more readily across languages than context-bound semantic knowledge, consistent with the RHM model (Kroll & Stewart, 1994; Kroll et al., 2010).

These patterns are consistent with previous bilingual research indicating that language experience modulates lexical performance. For instance, Sheng et al. (2013) found that Spanish-English bilingual children performed better in their dominant language and that lexical depth varied depending on language exposure. Similarly, our findings suggest that lexical accuracy is shaped not only by age but by relative proficiency and instructional context. Importantly, while children in higher grades demonstrate improved performance in both languages, the rate of improvement differed. In third grade, children in Study 1 had the highest performance accuracy, with English responses at 87% accuracy for both paradigmatic and syntagmatic associations, and Spanish responses at 70% and 63% accuracy, respectively. The convergence of English paradigmatic and syntagmatic accuracy at third grade likely reflects cumulative exposure to English academic language and structured literacy instruction, which strengthens both categorical and functional lexical associations. In contrast, the continued gap in Spanish suggests that while oral language use may support syntagmatic associations, limited literary academic exposure in Spanish may constrain the development of paradigmatic depth. Study 2 supports this interpretation, with older children more frequently using paradigmatic reasoning in English and thematic or functional strategies in Spanish, which demonstrates how language context shapes semantic processing.

Although the present results converge with prior work showing a developmental trend from syntagmatic to paradigmatic organization, the findings, particularly from the cognitive interviews, also indicate that these two associative modes can coexist and be flexibly activated depending on task demands and contextual cues. Rather than reflecting a complete replacement, the syntagmatic–paradigmatic shift may represent a gradual rebalancing between associative systems that continue to operate in parallel. This interpretation illustrates children’s capacity to draw on both relational types as needed, suggesting that lexical–semantic development involves dynamic interaction rather than a unidirectional progression.

### 4.2. Language Experience and Semantic Depth

Language exposure and dominance play a role in shaping children’s semantic depth. English associations reflected stronger paradigmatic accuracy, while Spanish associations more often relied on syntagmatic associations. This asymmetry suggests that children may rely on different association strategies depending on the depth of their semantic knowledge in each language. These findings are consistent with research showing that paradigmatic associations rely more heavily on academic vocabulary and literacy exposure, typically strengthened through formal education and reading (V. S. Cronin, 2002; Leider et al., 2013; Liu & Saad, 2025; Perfetti, 2007; Perfetti & Helder, 2022), whereas syntagmatic associations tend to reflect oral, everyday contexts, often grounded in home-based or conversational Spanish use (Peña et al., 2002, 2003; Sheng et al., 2006, 2013).

In Study 2, patterns in children’s responses aligned with a shift from syntagmatic to paradigmatic reasoning. Cognitive interviews suggested that paradigmatic strategies, such as categorization, synonymy, and antonymy were more commonly used in English, while Spanish responses more often reflected syntagmatic associations based on thematic or functional relationships. Our findings suggest that bilingual children flexibly adjust their reasoning strategies based on conceptual understanding and language experience. These patterns reflect not only relative dominance but also how each language is typically used, English often in academic and literate contexts, Spanish in conversational or home settings. Such context-dependent experience shapes which types of lexical associations transfer across languages, with conceptual links (e.g., categorization) generalizing more readily than context-bound ones. This distinction could explain why transfer of paradigmatic reasoning is more robust than transfer of syntagmatic associations (Kroll & Stewart, 1994; Kroll et al., 2010; McMillen et al., 2022).

While the quantitative and qualitative results differed in surface patterns of dominance, together they illustrate complementary dimensions of bilingual lexical development. Study 1 revealed group-level trends, showing greater paradigmatic accuracy in English and stronger syntagmatic associations in Spanish, reflecting differences in exposure and instructional experience. Study 2, in turn, illuminated the reasoning processes behind these patterns, how children actively navigated meaning across languages depending on task demands and familiarity. Viewed together, the two studies capture distinct yet converging levels of analysis: performance patterns at scale and conceptual depth through reflection.

The cross-language effect consistency aligns with the Interdependence Hypothesis (Cummins, 1979), which posits that conceptual knowledge transfers across languages. Children often applied reasoning developed in one language to interpret word associations in the other, particularly leveraging English-based semantic knowledge when responding in Spanish (Ordóñez et al., 2002). Thus, even when accuracy varied by language, the conceptual system supporting those associations appeared to be shared (Costa et al., 2023; Lemke et al., 2021). Our results also align with the Revised Hierarchical Model (RHM; Kroll & Stewart, 1994; Kroll et al., 2010), as English associations, likely shaped by greater exposure to print and formal instruction, reflected more abstract, paradigmatic reasoning, such as categorizing words by meaning or identifying synonyms and antonyms. In contrast, Spanish associations more often relied on syntagmatic reasoning, commonly developed through day-to-day oral language use and home-based interactions.

### 4.3. Advancing Understanding of the Syntagmatic-Paradigmatic Shift

Although the syntagmatic-paradigmatic shift has been an established topic in psycholinguistics (Brown & Berko, 1960; V. S. Cronin, 2002; Entwisle, 1966; Francis, 1972; McNeill, 1966; Nelson, 1977), most existing research is dated and based on monolingual, English-speaking samples. The classic view posits a linear shift from syntagmatic to paradigmatic associations around ages six to nine; however, our findings indicate that this shift is more gradual, context-dependent, and language-specific in bilingual children. In particular, the social, instructional, and linguistic experiences of bilingual children create a semantic landscape that is not easily captured by traditional models. For children in transitional bilingual programs, school instruction often begins at 90% Spanish and 10% English, but many children in our sample hear and use English at home. This asymmetry likely reflects differences in exposure: English, reinforced through literacy and schooling, supports more paradigmatic reasoning, whereas Spanish, used more in everyday contexts, retains stronger syntagmatic links.

The existing literature has emphasized production tasks (e.g., free word associations), overlooking comprehension-based measures (Hasan & Rahman, 2020; Read, 2008). Both of our studies offer a promising methodological contribution by examining lexical associations through a comprehension-based task presented in both auditory and visual modalities. The study design aligns more closely with the types of language processing required in school settings, where children must interpret and reason about word meanings during reading and listening activities (Elleman et al., 2009; Hwang et al., 2023; Liu & Saad, 2025; Zhang & Zhang, 2022). Given the rising number of bilingual students in U.S. classrooms, there is a critical need to revisit and update developmental models of lexical organization to reflect current sociolinguistic realities. Our sample, comprising bilingual children from diverse dual-language programs across multiple regions, demonstrates the linguistic variability that characterizes contemporary educational environments.

A key contribution of this study is the integration of accuracy-based measures with explanatory reasoning. Traditional word association research has typically treated responses as binary (correct/incorrect), offering limited insight into the processes that guide lexical access. By combining performance data (Study 1) with metacognitive reasoning (Study 2), our research emphasizes the importance of process-oriented approaches to semantic development. Children’s explanations reveal not only the depth of their conceptual understanding but also the strategies they used to navigate linguistic ambiguity and cross-language differences.

Our findings also offer practical implications for assessment and instruction. Teachers and clinicians working with bilingual children should consider not just whether a child produces a correct association, but how they arrive at their answer. Understanding the reasoning process behind lexical associations can help educators identify whether children are drawing on categorical or functional links and adjust instruction accordingly. For example, teachers might incorporate cross-language categorization activities, such as sorting words into semantic groups (e.g., animals, foods) across English and Spanish, or word-association discussions that invite students to explain why two words go together. These activities can strengthen semantic depth and promote flexible thinking about word relationships. Integrating such practices into dual-language literacy instruction, for instance, by comparing thematic vocabulary in both languages during shared reading, can support reading comprehension and vocabulary growth. In line with inclusive and differentiated instruction approaches (Andreou & Argatzopoulou, 2025; Nair et al., 2023), educators can adapt these strategies to each learner’s language profile and proficiency level, ensuring that bilingual children receive meaningful opportunities to engage with and connect concepts across their languages.

## 5. Limitations

These studies are not without limitations. First, some task items may have been too easy for bilingual children enrolled in dual-language programs, potentially reducing sensitivity to individual differences. In addition, the English and Spanish tasks were not in fully equivalent form, which may limit direct cross-linguistic comparisons. Future research should more clearly distinguish between syntagmatic and paradigmatic items in task design to avoid overlapping. Due to the study design, we did not include independent measures of vocabulary breadth and depth or detailed profiles of language use at home and school, both of which would provide richer insights into the relationship between exposure and semantic organization. In addition, because of the large-scale, classroom-level design and time constraints during school-based data collection, we were unable to gather detailed participant background questionnaires beyond age, grade, language, and instructional setting. Including such data in future studies could provide more precise insight into the type of bilingualism and its relation to lexical-semantic organization. Despite these limitations, both studies make an important contribution: they employ a novel comprehension-based task rather than production, demonstrating how bilingual children recognize and reason about word associations across languages.

## 6. Conclusions

The present studies offer new insights into the development of lexical-semantic associations in bilingual children by combining accuracy-based performance with qualitative reasoning data across two languages. Together, the quantitative and qualitative findings provide complementary perspectives on bilingual lexical development: Study 1 identified group-level patterns in syntagmatic and paradigmatic accuracy across English and Spanish, whereas Study 2 revealed the reasoning processes that underlie those patterns. The findings of these studies demonstrate how language experience and context shape the strategies children use to understand lexical relationships. This integration emphasizes the value of mixed-method approaches in capturing both the measurable outcomes and the conceptual depth of bilingual semantic development.

Building on these insights, both research and educational practice should continue exploring how instructional approaches can foster paradigmatic and syntagmatic associations. As mentioned, implementing cross-language semantic activities or bilingual word-mapping tasks may help children recognize deeper conceptual relationships between words. Such practices align with inclusive and differentiated models of instruction, which emphasize adapting learning experiences to students’ linguistic repertoires and developmental needs. Future work should examine how these patterns evolve in later elementary years and how instructional approaches, such as balanced literacy or content-integrated dual language models, support the development of both syntagmatic and paradigmatic associations. Longitudinal studies may also clarify how early lexical organization relates to broader academic outcomes, including reading comprehension and disciplinary language use across content areas.

## Figures and Tables

**Table 1:** Descriptive statistics of participant ages.

Group	M (SD)
Overall (*n* = 244)	7.87 (0.88)
Female (*n* = 133)	7.84 (0.91)
Male (*n* = 111)	7.91 (0.85)
Grade 1 (*n* = 86)	7.02 (0.53)
Grade 2 (*n* = 109)	8.05 (0.50)
Grade 3 (*n* = 49)	8.98 (0.51)

Note. *M* = mean; *SD* = standard deviation.

**Table 2:** Syntagmatic-paradigmatic proportion of items administered.

	Spanish		English	
Grade	PEP Para *M* (*SD*)	PEP Syn *M* (*SD*)	WMG Para *M* (*SD*)	WMG Syn *M* (*SD*)
First	0.55 (0.20)	0.55 (0.20)	0.79 (0.14)	0.77 (0.22)
Second	0.57 (0.18)	0.61 (0.21)	0.82 (0.12)	0.73 (0.24)
Third	0.63 (0.18)	0.70 (0.20)	0.87 (0.12)	0.87 (0.18)

Note. Para = Paradigmatic; Syn = Syntagmatic.

## Data Availability

The data presented in this paper is available on request from the authors. The data is not publicly available due to privacy concerns.

## Note

1 Although the sample for the cognitive-interview phase (*n* = 13) is relatively small, this is consistent with qualitative interview methodology in which thematic saturation rather than statistical generalization is the goal (see Hennink & Kaiser, 2022). Typical cognitive-interview studies reach theme saturation with approximately 5–7 participants per subgroup.

## Appendix A

**Table A1:** Anchor and Target Word Selection.

Tier (Anchor Word)	Description	Example Anchor Word	Target Word Relationship	Example Target Word
Tier I	Common, everyday words frequently encountered in spoken language.	Shoe	Synonym	Sneaker
		Happy	Antonym	Sad
		Dog	Thematic Relation	Leash
Tier II	Less frequent words that appear across multiple academic subjects.	Analyze	Synonym	Examine
		Contrast	Antonym	Similar
		Fair	Thematic Relation	Cotton candy
Tier III	Content-specific words found in specialized domains (e.g., science, math).	Circumference	Syntactic Relationship	Measure
		Chrysalis	Synonym	Cocoon
		Bird	Superordinate/ Subordinate	Eagle

Note. Tier I words are commonly encountered in everyday speech, Tier II words appear across academic disciplines, and Tier III words are content-specific. Target words were chosen based on their relationship to the anchor word, ensuring linguistic diversity across items. The complete word list is not included because it contains proprietary materials and components that are still under development as part of an ongoing assessment validation project.

## Appendix B

**Table A2:** Sample of Cognitive Interview Responses.

Association Type	Participant Categorization	Words Selected	Participant Interpretation	Response Excerpt
Paradigmatic	Paradigmatic	rail, bleacher	size, theme, synonym	“*Because there are two definitions of rail so that’s why some other people might choose beach but this definition of rail for me is the rail that you see at theme parks where you can hold on to something while you’re walking and sometimes they have those at bleachers during a football game or a baseball game and that’s my experience.*”
Paradigmatic	Paradigmatic	aquarium, tank	size, theme	“*Because an aquarium is a tank full of fish and other creatures and a tank is just the empty version of it with just water and sand at the bottom or sometimes pebbles at the bottom*.”
Paradigmatic	Paradigmatic	bien, mal (good, bad)	antonym	*“So bien vamos a decir que está feliz pero mal es como que estás triste enojado so es como lo opposite” (So let’s say he’s happy but bad is like being sad or angry so it’s like the opposite).*
Paradigmatic	Syntagmatic	alumno, lápiz (student, pencil)	mutually dependent, semantic	“*Porque los alumnos usan lápices para escribir y para dibujar*” (“Because students use pencils to write and to draw”).
Syntagmatic	Syntagmatic	reloj, tiempo (clock, time)	Mutually dependent, theme	“*Porque el reloj te dice el tiempo porque el reloj puede saber el tiempo porque está programada para hacer eso*” (“Because the clock tells you the time because the clock can know the time because it is programmed to do that.”
Paradigmatic	Syntagmatic	discussion, silence	mutually dependent, theme	“*Because in a discussion when someone is talking you have to be silent.”*

Note. Words selected show the specific word pair chosen during the paper-based format of the task. Participant interpretation reflects the reasoning or relational theme based on participant responses. Response excerpt provides a representative quotation from various cognitive interviews.
